# Rose Bengal Crosslinking to Stabilize Collagen Sheets and Generate Modulated Collagen Laminates

**DOI:** 10.3390/ijms21197408

**Published:** 2020-10-08

**Authors:** Stefanie Eckes, Joy Braun, Julia S. Wack, Ulrike Ritz, Daniela Nickel, Katja Schmitz

**Affiliations:** 1 Clemens-Schöpf-Institute of Organic Chemistry and Biochemistry, Technical University of Darmstadt, Alarich-Weiss-Straße 8, 64287 Darmstadt, Germany; stefanie.eckes@tu-darmstadt.de (S.E.); wack.juliasusanne@googlemail.com (J.S.W.); 2 Department of Orthopaedics and Traumatology, BiomaTiCS, University Medical Center, Johannes Gutenberg University, Langenbeckstraße 1, 55131 Mainz, Germany; joybraun@uni-mainz.de (J.B.); ritz@uni-mainz.de (U.R.); 3 Berufsakademie Sachsen—Staatliche Studienakademie Glauchau, University of Cooperative Education, Kopernikusstraße 51, 08371 Glauchau, Germany

**Keywords:** collagen type I, rose bengal and green light crosslinking, swelling degree, mechanical stability, drug release, vancomycin

## Abstract

For medical application, easily accessible biomaterials with tailored properties are desirable. Collagen type I represents a biomaterial of choice for regenerative medicine and tissue engineering. Here, we present a simple method to modify the properties of collagen and to generate collagen laminates. We selected three commercially available collagen sheets with different thicknesses and densities and examined the effect of rose bengal and green light collagen crosslinking (RGX) on properties such as microstructure, swelling degree, mechanical stability, cell compatibility and drug release. The highest impact of RGX was measured for Atelocollagen, for which the swelling degree was reduced from 630% (w/w) to 520% (w/w) and thickness measured under force application increased from 0.014 mm to 0.455 mm, indicating a significant increase in mechanical stability. Microstructural analysis revealed that the sponge-like structure was replaced by a fibrous structure. While the initial burst effect during vancomycin release was not influenced by crosslinking, RGX increased cell proliferation on sheets of Atelocollagen and on Collagen Solutions. We furthermore demonstrate that RGX can be used to covalently attach different sheets to create materials with combined properties, making the modification and combination of readily available sheets with RGX an attractive approach for clinical application.

## 1. Introduction

Collagen type I is the most frequently used natural biomaterial for regenerative medicine and tissue engineering since, as the main component of the extracellular matrix, it is biocompatible and biodegradable [1,2,3,4]. Collagen may be extracted from animal tissue [5,6] or produced recombinantly [7,8]. To reduce the antigenicity of collagen type I, the terminal telopeptides of collagen can be removed enzymatically to obtain atelocollagen [9,10]. While the native form of collagen type I is crosslinked intra- and intermolecularly providing tensile stiffness [11,12], the processed form after extraction lacks mechanical strength. To be applied in tissue engineering, extracted collagen type I and atelocollagen need to be crosslinked to increase mechanical strength and enzymatic resistance [9,11]. There are chemical, physical and biological methods available to improve collagen properties. Chemical crosslinking is mostly accomplished by glutaraldehyde [13,14,15], ethyl-3(3-dimethylamino) propyl carbodiimide/ N-hydroxysuccinimide (EDC/NHS) [11,16,17], diisocyanates [18,19] or oxidized sugar [20,21]. However, chemical crosslinking is often time consuming due to the required wash steps, potentially leaves toxic residues and may reduce biocompatibility [18,22]. Some crosslinkers like glutaraldehyde may also be cytotoxic and lead to calcification [18,23]. For physical crosslinking, dehydrothermal treatment (DHT) [24,25] and UV irradiation [10,26,27] are commonly used. However, these methods can lead to partial degradation of collagen and to protein denaturation [24]. An alternative photochemical approach called rose bengal and green light crosslinking (RGX) uses innocuous visible light for the activation of rose bengal (RB) in order to modify collagen [28,29,30,31,32,33,34]. RB is a well-known agent for the repair of corneal damage [34,35] and has been approved for several clinical applications [36,37,38]. Studies have shown that RGX can be used to stiffen the cornea [34] and to repair corneal damage [39], to bond skin grafts for surgical repair [30] and to treat disc degeneration [40]. Crosslinking of unfunctionalized collagen by RGX has been shown to improve thermostability and mechanical properties and to modify swelling ratio of collagen membranes and drug release [31,32,33].

In these studies, collagen membranes are commonly produced under laboratory settings, and parameters are optimized to produce materials with properties tailored to one specific application. In clinical practice, it is desirable to use commercially available collagen sheets and tailor their properties to the respective application with a simple procedure.

**Figure 1:**
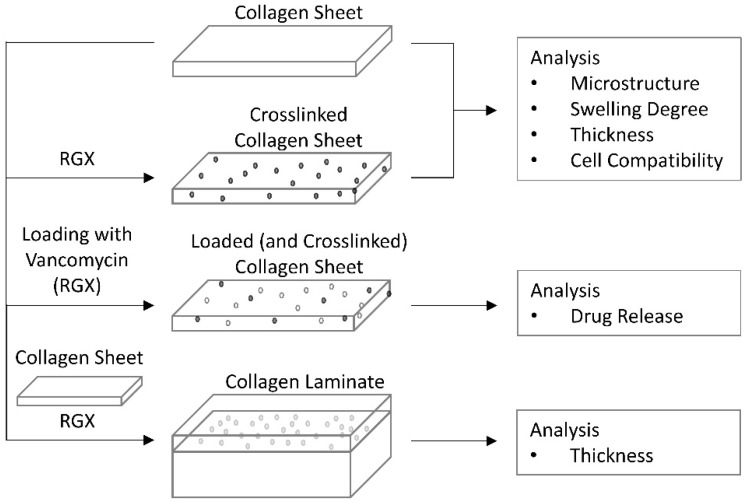
Overview of methods used for collagen analysis.

We assessed three commercially available bovine collagen type I materials with different thicknesses, densities and crosslinking degrees that may be used as wound inserts for drug delivery. To characterize these materials, we investigated their swelling degree, microstructure, mechanical stability and release of vancomycin as a model drug. We examined to what extent these properties could be modified by RGX. The use of an inexpensive light source such as a power LED makes this approach suitable for a clinical setting. We also tested whether the applied RB concentrations were tolerated by different cells and examined the cell behavior on unmodified and modified collagen samples. Moreover, we demonstrated that RGX can be used to crosslink different commercially available collagen sheets to obtain anisotropic materials that combine the mechanical properties of the starting materials (Figure 1).

## 2. Results

Unless stated otherwise, all experiments were performed in triplicates for each tested collagen sample.

### 2.1. Collagen Sheets—An Overview

**Figure 2:**
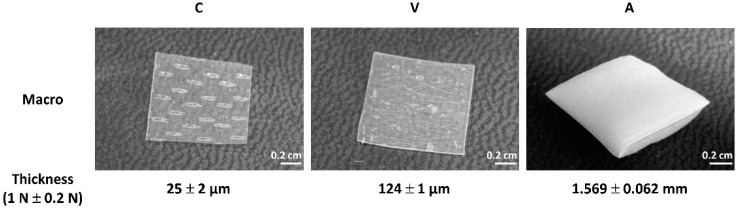
Photographs of collagen sheets and their thickness as analyzed via height gauge. Sample size: 1 × 1 cm^2^. (**C**) Collagen Solutions, (**V**) Viscofan and (**A**) Atelocollagen.

The thickness of the three different commercially available collagen sheets (Collagen Solutions, Viscofan and Atelocollagen) was measured in their dry state as received via height gauge under force application. Collagen Solutions collagen (C) is a thin collagen sheet with discernible imprints with a thickness of 25 ± 2 µm (Figure 2). Viscofan (V), is a more compact collagen sheet with a thickness of 124 ± 1 µm. Atelocollagen (A) is a sponge-like collagen with a thickness of 1.569 ± 0.062 mm.

### 2.2. Swelling Degree and Microstructure of Collagen Sheets

Swelling degree measurements were carried out to characterize the collagen sheets and to determine the loading capacity for drug release. The microstructure was analyzed by transmitted light microscopy (TLM) and scanning electron microscopy (SEM).

#### 2.2.1. Collagen Sheets as Received

**Figure 3:**
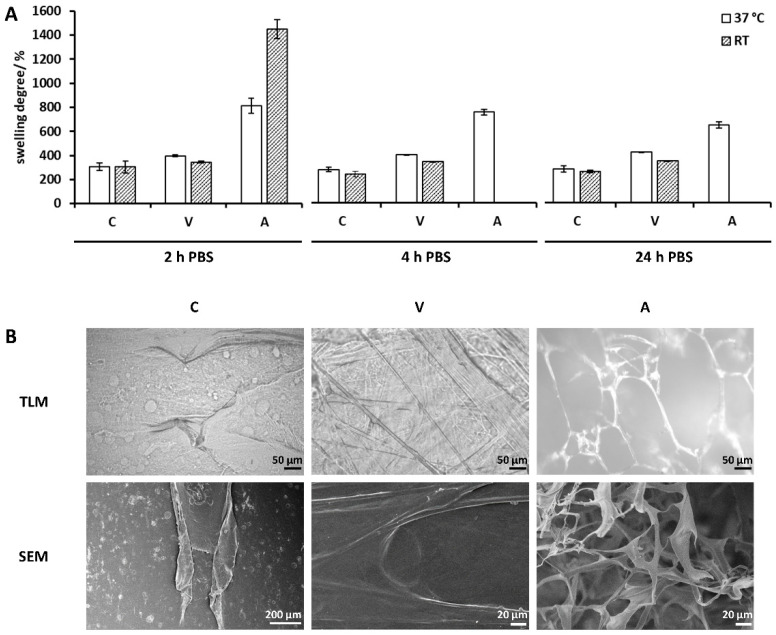
Swelling behavior and microstructure of collagen sheets. (**A**) Swelling degree of collagen sheets “as received” at 37 °C and RT after 2 h, 4 h and 24 h in relation to their dry weight (100%). (**B**) transmitted light microscopy (TLM) and SEM images of collagen sheets. Samples analyzed via TLM were conditioned in phosphate-buffered saline (PBS). TLM magnification: 200×. SEM magnification: V and A: 1000×, C: 200×. C: Collagen Solutions, V: Viscofan and A: Atelocollagen.

The swelling degree of collagen samples “as received” was measured after 2 h, 4 h and 24 h swelling in 2 mL of phosphate-buffered saline (PBS) at 37 °C and room temperature (RT) (Figure 3A). After 2 h, swelling in PBS was completed. For Collagen Solutions, no temperature dependency was observed, and collagen sheets showed an equal swelling degree of 300% (w/w). Swelling degrees of 400% (w/w) and 340% (w/w) were observed for Viscofan collagen sheets at 37 °C and RT, respectively, indicating a temperature dependency. A significant temperature dependency was observed for Atelocollagen samples. Samples reached 810% (w/w) of their dry mass at 37 °C and up to 1440% (w/w) at RT. The swelling degree after 4 h and 24 h could only be measured at 37 °C, since samples at RT were destroyed during measurement after 2 h due to instability of the collagen sponge. The loading capacity was determined after 2 h, as swelling was completed, and used as the buffer volume for collagen sheet loading. For RGX, samples were loaded with RB, crosslinked and dried prior to further experiments. To assess the effect of loading and drying on swelling properties, collagen sheets were loaded with PBS, lyophilized and then analyzed. In case of Atelocollagen, this pretreatment led to a lower swelling degree and to a loss of temperature dependency but to no stability increase (Supporting Information (SI), Figure S1). These samples are further referred to as “unmodified” in order to distinguish them from samples “as received”.

Collagen samples were further characterized by TLM and SEM (Figure 3B). The samples of Collagen Solutions appeared to be relatively dense with no detectable fiber structure. Viscofan samples, however, showed a highly crosslinked structure with long fibers that were discernible by light microscopy as well as by SEM. Samples of Atelocollagen showed an open, sponge-like structure with wide pores visible by light microscopy in the wet state. The structure appeared more dense by SEM imaging in the dry state.

#### 2.2.2. Modification of Collagen Sheets by RGX

In order to obtain stabilized collagen sheets for improved handling, samples were treated with RGX under different conditions. Concentrations of 0.1% RB and 0.01% RB were combined with exposure times of 10 min and 60 min, respectively. A concentration of 0.1% RB had a toxic effect on osteoblasts (data not shown) and was therefore considered unsuitable. Exposure of collagen sheets for 60 min led to the same effect as an exposure time of 10 min (data not shown). Therefore, 0.01% RB and 10 min green light exposure were selected.

**Figure 4:**
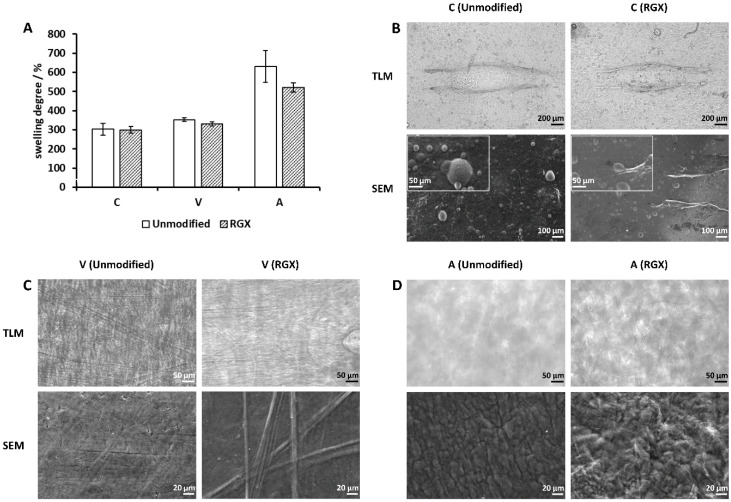
Impact of rose bengal and green light collagen crosslinking (RGX) on swelling degree and microstructure. (**A**) Swelling degree of unmodified and modified (RGX: 10 min, 0.01% rose bengal (RB)) collagen sheets after 2 h at 37 °C in relation to their dry weight (100%). (**B**–**D**) TLM and SEM images of collagen sheets before (unmodified) and after RGX (10 min, 0.01% RB). Samples analyzed via TLM were conditioned in PBS. (**B**) Collagen Solutions. TLM magnification: 50×. SEM magnification: 200× and 1000× (inset). (**C**) Viscofan. TLM magnification: 200×. SEM magnification: 1000×. (**D**) Atelocollagen. TLM magnification: 200×. SEM magnification: 1000×; C: Collagen Solutions, V: Viscofan and A: Atelocollagen.

Collagen sheets of Collagen Solutions, Viscofan and Atelocollagen were modified by RGX, and their 2 h swelling degree was analyzed. Swelling analysis was carried out at 37 °C since loaded Atelocollagen sheets had shown no temperature dependency, and a higher swelling degree had been recorded for loaded sheets of Collagen Solutions and Viscofan at 37 °C so that an influence by RGX would be easier to detect. For Collagen Solutions and Viscofan samples, swelling behavior was independent of RGX (Figure 4A). In the case of Collagen Solutions sheets, unmodified and modified samples equally reached 300% (w/w) of their dry mass in the swollen state. Viscofan samples showed a swelling degree of 330% (w/w) for unmodified samples and 350% (w/w) for modified samples. However, the swelling degree of Atelocollagen decreased from 630% (w/w) to 520% (w/w) upon modification. Furthermore, Atelocollagen sheets showed a more compact structure after RGX and were stable during the measurements, which facilitated handling.

In the case of Collagen Solutions sheets, no changes in microstructure between unmodified and modified samples were detectable (Figure 4B). Viscofan samples showed no structural changes in TLM, however, analyzed by SEM, fibers in collagen samples after RGX appeared more pronounced (Figure 4C). Light microscopy and SEM of unmodified Atelocollagen samples displayed a collapse of the open, sponge-like structure seen in samples “as received”. Furthermore, unmodified samples appeared diffuse by light microscopy, while fibers were discernible after RGX. SEM images of unmodified Atelocollagen samples showed an aggregation of fibers, whereas modified samples showed a more voluminous arrangement (Figure 4D).

### 2.3. Thickness Analysis of Collagen Sheets

**Figure 5:**
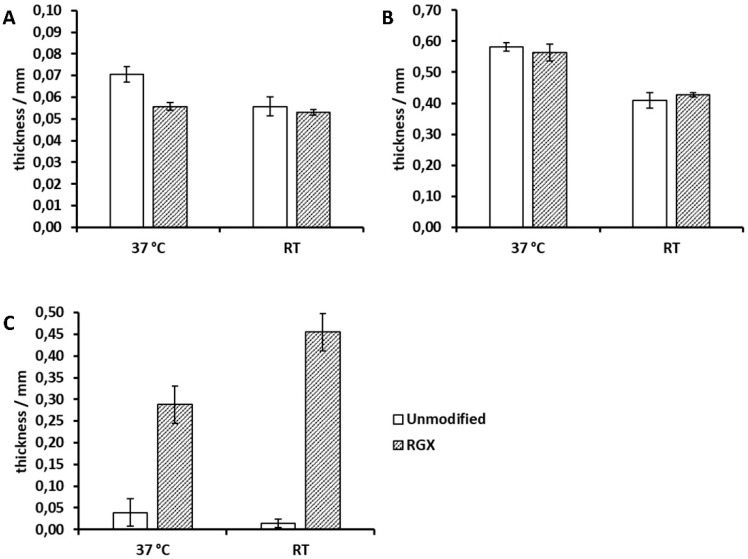
Thickness of unmodified and modified (RGX: 10 min, 0.01% RB) collagen sheets at 37 °C and RT. Samples were conditioned in PBS for 24 h before measurement. (**A**) Collagen Solutions, (**B**) Viscofan, (**C**) Atelocollagen.

To assess the effect of RGX on stability (particularly for Atelocollagen), mechanical behavior was investigated. For this purpose, the thickness of unmodified and modified collagen sheets was measured at 37 °C and RT via height gauge under force application as a measure of mechanical stability. In the case of Collagen Solutions sheets, RGX caused a decrease in thickness of 21%, thus revealing an effect not seen in swelling degree measurements (Figure 5A). Furthermore, the thickness of unmodified samples decreased by 21% from 37 °C to RT, indicating a temperature dependency. However, RGX led to a loss of the temperature dependency manifesting as equal thicknesses at 37 °C and RT. This effect was not seen in Viscofan samples, since their thickness remained temperature dependent after modification (Figure 5B). RGX of Viscofan sheets showed no influence on thickness. In case of Atelocollagen sheets, unmodified samples were irreversibly compressed after the first or second measurement, which resulted in small thickness values (Figure 5C). RGX of Atelocollagen sheets led to a significant increase in their thickness, indicating a higher mechanical stability and, thus, confirming the results of swelling degree measurements. This effect was greater at RT than at 37 °C, indicating a temperature dependency not measurable for unmodified samples.

### 2.4. Cell Viability on Collagen Sheets

**Figure 6:**
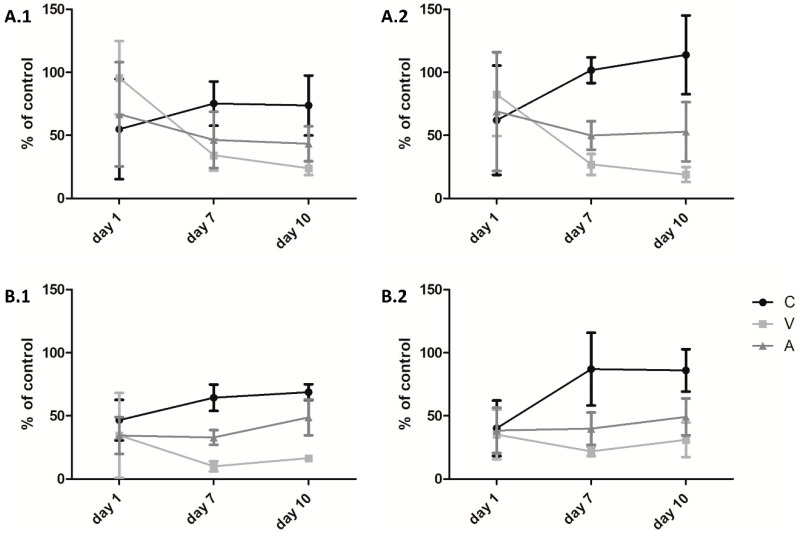
Proliferation of (**A**) human osteoblasts and (**B**) primary human muscle cells on (1) unmodified and (2) modified (RGX: 10 min, 0.01% RB) collagen sheets for 1, 7 and 10 days. The results are presented in percentage of the control without collagen sheet (100%). C: Collagen Solutions, V: Viscofan and A: Atelocollagen.

To examine cell behavior, primary human osteoblasts and primary human muscle cells were seeded on unmodified sheets, sheets treated with RGX and the culture plate alone as control. Cell viability was measured on days 1, 7 and 10 (Figure 6). In general, no differences were observed on day 1, whereas on day 7, clear differences were detected. Viability increased from Viscofan to Atelocollagen to Collagen Solutions samples. There was a manifestation of this trend on day 10. Crosslinked Collagen Solutions sheets led to an increase in cell proliferation in muscle cells as well as in osteoblasts. In the latter, viability exceeded 100%, demonstrating a better proliferation on the crosslinked sheets than on the plain culture dish. Proliferation of both cell types on Atelocollagen samples started to increase distinctly after 7 days. As with Collagen Solutions samples, osteoblast viability was higher on crosslinked sheets, while no difference was seen in muscle cells. Viability of the cells decreased from day 1 to day 7 on Viscofan sheets, and only a very small proliferation was measured during the following days.

### 2.5. Release of Vancomycin

**Figure 7:**
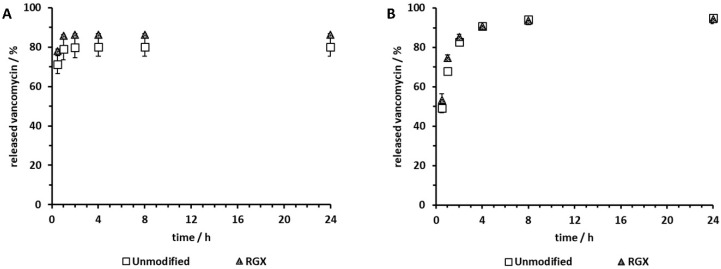
Release of vancomycin from unmodified and modified (RGX: 10 min, 0.01% RB) collagen samples over the course of 24 h. Collagen sheets were loaded with 1000 µg vancomycin (100%). (**A**) Collagen Solutions, (**B**) Atelocollagen.

To evaluate the impact of RGX on collagen sheet-based drug delivery, the release of vancomycin from both unmodified and modified collagen samples was quantified via HPLC. Since Viscofan sheets were unsuitable for the proliferation of human osteoblasts and human muscle cells, the release of vancomycin was determined only for Collagen Solutions and Atelocollagen samples. Modification of vancomycin-loaded Collagen Solutions sheets by RGX did not affect the release profile (Figure 7A). Vancomycin was rapidly released within the first 2 h. Within 30 min, 71% ± 5% and 78% ± 1% of the loaded vancomycin was released from unmodified and modified samples, respectively. Likewise, RGX of vancomycin-loaded Atelocollagen samples showed no impact on the release profile (Figure 7B). After an initial burst in which half of the loaded amount was released within 0.5 h, vancomycin release was still observed after 24 h. However, the entire amount of loaded vancomycin could be recovered from neither unmodified nor modified samples.

### 2.6. Thickness Analysis of Collagen Laminates

**Figure 8:**
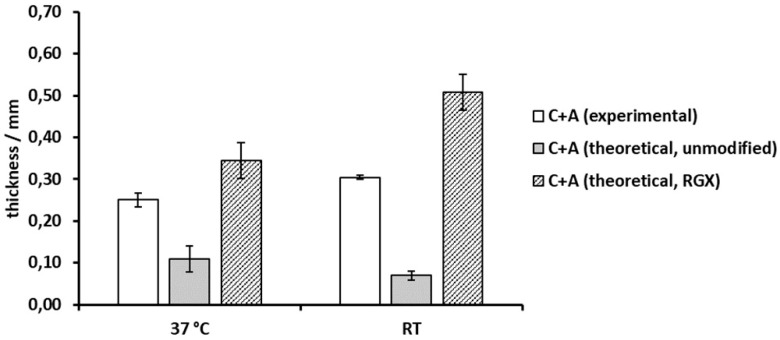
Thickness of collagen laminates consisting of Collagen Solutions collagen (C) and Atelocollagen (A) prepared by RGX (10 min, 0.1% RB) compared to their theoretical thickness (sum of thicknesses of collagen single sheets) of unmodified and modified samples (RGX) at 37 °C and RT. Samples were conditioned in PBS for 24 h before measurement.

Each collagen sheet had shown different characteristics. In order to combine features of different collagen sheets, collagen laminates consisting of two collagen sheets were generated. These laminates were analyzed via height gauge under force application to assess thickness and mechanical stability as exemplary properties. The thickness of the collagen laminates consisting of Collagen Solutions collagen and Atelocollagen was compared to their theoretical thickness as calculated from single sheet measurements of unmodified and modified samples at 37 °C and RT, respectively (Figure 8). Crosslinking of Collagen Solutions and Atelocollagen sheets led to significantly increased thickness of 130% and 340% compared to theoretical thickness of unmodified samples at both 37 °C and RT. In comparison to modified sheets, the collagen laminate reached 75.2% and 61.2% of the theoretical thickness at 37 °C and RT, respectively. Furthermore, the thickness of the laminate showed a slight temperature dependency.

## 3. Discussion

### 3.1. Microstructure, Swelling Degree and Mechanical Properties of Collagen Sheets

Measurements of thickness via height gauge under force application of the different collagen samples in their dry state revealed that the Collagen Solutions sample was the thinnest collagen sheet with a thickness of 25 ± 2 µm, compared to Viscofan and Atelocollagen samples which were 5 times and 62 times thicker, respectively. The highest compression to approximately one third of its original thickness could be measured for Atelocollagen, due to its open sponge-like structure seen under TLM and SEM (Figure 2, Figure 3B). Thickness measurements of samples “as received” and unmodified collagen samples showed that loading of collagen sheets with PBS had no impact (SI, Figure S3).

We used thickness analysis under force application also to investigate changes in mechanical stability at different temperatures before and after crosslinking. This reflects the deformation of the material during gentle handling that may drive out liquid from the hydrogel or even lead to irreversible compression if stability is insufficient. It is therefore well suited to assess the effect of crosslinking on mechanical stability to improve handling. We also characterized all materials by swelling analysis as a measure of rigidity.

During swelling degree analysis at RT and 37 °C, all samples “as received” showed a significant mass increase. For Collagen Solutions samples, the mass increase during the entire measurement period of 24 h was temperature independent. This result was unexpected, because a temperature dependency has been described for the swelling degree of collagenous materials. Zhao et al. [41] reported a higher swelling ratio at 37 °C compared to 4 °C for human-like collagen hydrogels crosslinked with transglutaminase. Nagorski et al. [42], however, reported a decrease in swelling with an increase in temperature for collagen type I. Since Collagen Solutions samples were very thin and already highly crosslinked (Figure 2, Figure 3B), we assumed that differences in absorbed volumes were too small to be measurable via analytical balance. Thickness analysis, however, revealed a 25% higher thickness of unmodified Collagen Solutions samples at 37 °C compared to RT. After RGX, this temperature dependency was lost (Figure 5A).

Viscofan samples possessed a highly compact structure and long fibers (Figure 3B). Due to the higher thickness of Viscofan sheets compared to samples of Collagen Solutions, a temperature dependency was measurable as a 15% increase in swelling degree (Figure 3A) and a 40% higher thickness (Figure 5B) at 37 °C compared to RT. This observation supports the findings of an increase in swelling ratio with temperature by Zhao et al. [41]. They explain this effect by a strengthening of hydrophilic interactions at elevated temperatures so that water interacts more readily with hydrophilic groups in the collagen matrix, leading to an expansion of the hydrogel. We furthermore assume that hydrogen bonds can more easily break and re-form at elevated temperatures, so that cavities within this dense collagen material can enlarge and entrap more PBS, leading to higher swelling degrees and thicknesses. RGX of Viscofan sheets had no impact on swelling degree and thickness analysis. This was to be expected since the microstructure of this material appeared very dense in the unmodified state (Figure 3B).

Atelocollagen “as received” was the thickest collagen sample and revealed the highest swelling degree and temperature dependency of all collagen samples, with a swelling degree at RT almost twice as high as at 37 °C. Large swelling ratios of Atelocollagen have been reported previously [43,44]. As revealed by light microscopy and SEM, Atelocollagen possessed much larger cavities than Collagen Solutions and Viscofan samples (Figure 3B). In line with the explanation for Viscofan samples, the faster exchange of hydrogen bonds at higher temperatures could enlarge the cavities of Atelocollagen to an extent at which the capillary effect is reduced. Consequently, PBS was less well contained, meaning that removal of non-absorbed liquid before the measurement would also remove buffer from within the collagen sponge. This could explain the observed lower swelling degrees at 37 °C compared to RT. For Atelocollagen samples, loading with PBS and drying (to obtain “unmodified” samples) had a significant impact in that samples lost their temperature dependency and swelling decreased by a factor of 1.74 and 2.4 compared to samples “as received” at 37 °C and RT, respectively (SI, Figure S1). This is probably due to the collapsed open sponge-like structure (Figure 4D) that we observed after loading with PBS and due to the higher ionic strength within the sample [45,46]. Atelocollagen samples “as received” and unmodified Atelocollagen samples were very instable during general handling and swelling degree measurements as a result of the open sponge-like structure. This observation was further supported by thickness analysis, in which unmodified samples were irreversibly compressed after the first or second measurement, which resulted in thickness values that were even two to four times smaller than samples of the thin Collagen Solutions film. RGX had the highest impact on the Atelocollagen samples with significant changes in both, swelling degree and thickness. While the swelling degree at 37 °C was reduced by almost 20% (Figure 4A), the thickness of modified samples was 7.4 and 32.7 times higher at 37 °C and RT (Figure 5C), respectively. This demonstrates a remarkable stability increase after crosslinking, which was also noticeable during handling. This is furthermore in agreement with previous reports about crosslinking experiments using other common methods to assess stability changes. Vizárová, Bakoš, Rehakova and Macho [47] modified atelocollagen by UV irradiation and chemical crosslinking, leading to lower swelling ratios compared to the unmodified control. Measurements of the ultimate tensile stress (UTS) revealed a higher mechanical strength of atelocollagen. These findings are supported by an investigation of Rousseau and Gagnieu [21] in which crosslinking of atelocollagen by oxidized sugar led to a decrease in the swelling ratio and a stability increase measured via differential scanning calorimetry (DSC). Shrestha, Hamblin and Kishen [48] reported a significant increase in toughness for dentin collagen crosslinked by RGX. Chan and So [31] measured higher stabilities after RGX of collagen hydrogels by DSC and tensile testing. They also found a reduction in swelling degree accompanied by reduced pore volumes. Atelocollagen treated with RGX imaging via light microscopy and SEM showed the development of a fibrous structure, while large pores were no longer discernible. This is in accordance with a reduced pore volume and the reduction in swelling degree (Figure 4A). Interestingly, we have observed both of these effects as well as a significantly higher thickness for Atelocollagen samples in a control set that was treated with RB only (SI, Figure S2, SI Figure S4). A reduction in swelling degree was also seen for Collagen Solutions samples in this control, suggesting that ambient light exposure is sufficient for crosslinking (SI, Figure S2). This effect was also described by Chan and So [31]. A repetition of this control experiment with RB under exclusion of stray light led to thicknesses comparable to unmodified collagen samples. Exposure of Atelocollagen samples to green light without RB had no impact on thickness.

Collagen hydrogels have been crosslinked by a variety of methods to improve mechanical stability. Apart from RGX [31,48] and crosslinking by oxidized sugar [21], collagen has been crosslinked by other reagents such as EDC/NHS, glutaraldehyde or microbial transglutaminase [49,50]. Using bireactive small molecules requires extensive washing to avoid toxic effects, while enzymes for crosslinking may lose activity during storage. RGX is a robust method that requires few handling steps starting from dry collagen sheets “as received” and leads to a significant increase in mechanical stability in soft materials such as Atelocollagen.

### 3.2. Cell Viability on Collagen Sheets

Human osteoblasts and human muscle cells reacted almost identically to the different collagen sheets. The most pronounced proliferation was measured on Collagen Solutions samples. The proliferation of the osteoblasts on crosslinked sheets was higher than on unmodified control sheets. On day 1 and 7, no proliferation (osteoblasts) or minor proliferation (muscle cells) was measured on Atelocollagen sheets, while proliferation increased from day 7 to 10. The sponge-like structure could be a reason for this behavior since the cells require more time to form cell-cell contacts inside the three-dimensional matrix. The same results were obtained with a threefold higher amount of cells (data not shown). Therefore, we assume that the number of cells was still too low, or cells needed more time to acclimate on the Atelocollagen sponge. On Viscofan sheets, proliferation was negligible over the whole time of experiment. The very compact structure could hinder the adhesion and migration of the cells into the material. Higher viability was detected particularly on crosslinked Atelocollagen and Collagen Solutions samples. The increase in mechanical stability and rigidity due to RGX could be a potential reason for the improved proliferation of osteoblasts since bone is a mechanically stable compartment of the body. Our cell culture results strongly differ from the study of Rothamel et al. [51], in which they tested four commercially available collagen membranes with periodontal ligament fibroblasts and SaOs-2. The cells’ density and proliferation capacity on the different scaffolds was extremely low when compared to the culture plate. On the contrary, in our investigations, the viability of cells especially on Collagen Solutions membranes was comparable or even better than on the culture plate alone. Nevertheless, due to the different conditions (cells, culture period, etc.) these studies are hard to compare. It has to be kept in mind that the surface morphology of the used materials affects, for example, the attachment, proliferation and cytokine production. Therefore, it is generally hard to compare results when different biomaterials are used [52].

### 3.3. Release of Vancomycin

Vancomycin was released rapidly from unmodified Collagen Solutions sheets, which was to be expected, as these sheets are very thin and therefore diffusion paths are short. The release from Atelocollagen was somewhat prolonged with half of the vancomycin released after 30 min compared to approximately two thirds released from Collagen Solutions samples over the same time. Since Atelocollagen sheets are 62 times thicker and more porous than those of Collagen Solutions, diffusion paths are longer; thus, a prolonged release was expected. According to Rosenblatt et al. [53], the molecular weight of active agents has to be very large (200–300 kDa) in order to achieve hindered diffusion. Since vancomycin has a molecular weight of only 1.5 kDa, no hindered diffusion was to be expected. Singh et al. [54] demonstrated that active agents were released much slower from underloaded matrices compared to those loaded to capacity or overloaded. However, vancomycin has not been observed to bind to collagen [55], meaning that the loading capacities for vancomycin were well exceeded by the amounts used in our study, and no sustained release was to be expected. In all of our experiments only 80–90% of the vancomycin could be recovered after 24 h. This could not be attributed to vancomycin interaction with the used plasticware (data not shown). We therefore assume that a fraction of vancomycin still interacts with the collagen matrix to be released at a slow rate in amounts below the detection limit.

RGX has been described to reduce the initial burst during the release of bovine serum albumin (BSA) from collagen hydrogels by B. Chan et al. [32]. However, BSA is a 66.5 kDa negatively charged protein and therefore likely to bind to positively charged collagen at pH 7.4 [56]. It is also well-known to interact with small molecules such as RB [57]. It is therefore likely that BSA is covalently linked to the collagen matrix during RGX, leading to kinetics that are not only controlled by diffusion. In our experiments, the kinetics and the maximum amount of the released compound were not influenced by RGX in either material, indicating that there was no covalent crosslinking of vancomycin to the collagen matrix.

### 3.4. Collagen Laminates

In a clinical setting, commercially available collagen sheets may be combined in situ to tailor their properties to different applications, e.g., Atelocollagen that supports cell growth may be combined with Collagen Solutions sheets to obtain a more stable material. In multilayer films reported in the literature, the carrier material is often already provided in a composite [58,59,60,61]. Furthermore, multilayer films have been generated, e.g., by UV irradiation in the presence of a photoinitiator [62], or are held together by electrostatic interactions of polyelectrolytes [61,63]. To our knowledge, we are the first to report a modular generation of collagen laminates by RGX that can be carried out in situ. Covalently linking layers of collagen materials can facilitate handling and prevent slippage. As proof of concept, unmodified sheets of Collagen Solutions and Atelocollagen were crosslinked by RGX and analyzed via height gauge. The collagen laminate was two and four times thicker than the theoretical thickness calculated from unmodified single sheets at 37 °C and RT, respectively. As irreversible compression led to small thickness values for unmodified Atelocollagen, this indicates that Atelocollagen is stabilized by laminate formation with Collagen Solutions samples. However, compared to the theoretical thickness calculated from modified single sheets, the laminate thickness was 24.8% (37 °C) and 38.8% (RT) lower. This can be explained by differences in sample preparation. For RGX of single sheets, dry samples were loaded with RB for 2 h, resulting in an even distribution of RB, while in the case of collagen laminates, RB was pipetted onto the surface of a PBS-loaded collagen sheet directly before light exposure. Thus, only the contact area could be crosslinked, leaving remote parts of the collagen sponge unmodified and prone to compression during measurement. In agreement with the theoretical thickness calculated from modified collagen sheets, the collagen laminate was thicker at RT than at 37 °C. Addition of the thicknesses from single sheet measurements of unmodified samples had predicted the opposite due to the irreversible compression of the Atelocollagen samples in single sheet measurements (Figure 8).

## 4. Materials and Methods

### 4.1. Collagen Sheets

Non-perforated collagen film (“Collagen Solutions”) was purchased from Collagen Solutions (London, UK). “Collagen membrane” was purchased from Viscofan BioEngineering (Weinheim, Germany) and is further referred to as “Viscofan”. Atelocollagen sponge (CLS-01, Koken Co. Ltd., Tokyo, Japan) is a high purity bovine dermis-derived porous, sponge-like collagen that is designed for three-dimensional cell culture and is referred to as “Atelocollagen”.

### 4.2. Collagen Preparation

Collagen sheets were cut to 1 × 1 cm^2^ squares or to circles with a diameter of 1 cm, respectively. Unloaded collagen samples are referred to as “as received”. If not mentioned otherwise, “modified” collagen samples were treated by RGX, “unmodified” collagen sheets were loaded only with PBS (137 mM NaCl, 2.7 mM KCl, 1.5 mM KH_2_PO_4_, 8.1 mM Na_2_HPO_4_, pH 7.4) and used as control, and samples were lyophilized after treatment.

### 4.3. Analysis of Collagen Swelling Degree

Collagen sheets of the size 1 × 1 cm^2^ were weighed to obtain the dry weight (m_d_). Samples were incubated in 2 mL of PBS at RT and at 37 °C. The weight of the wet collagen sheets (m_w_) was measured after 2 h, 4 h and 24 h of incubation after blotting non-absorbed liquid. The swelling degree (1) and capacity (2) were calculated as follows:

Swelling degree = m_w_ ∙ m_d_^−1^ ∙ 100% (1)

Capacity (per cm^2^) = (m_w_ − m_d_) ∙ *ρ* (PBS)^−1^ (2)

### 4.4. Collagen Loading

For loading, collagen sheets of the size of 1 × 1 cm^2^ were swollen in a Petri dish (Sarstedt, Nümbrecht, Germany) for 2 h with a buffer volume corresponding to their capacity determined by swelling degree measurements. The capacity of round sheets was calculated from the buffer capacity of square sheets (1 × 1 cm^2^). Depending on the investigation, samples were loaded with PBS, vancomycin (Carl Roth GmbH, Karlsruhe, Germany) in PBS, rose bengal (Alfa Aesar, Kandel, Germany) in PBS or a combination of both.

### 4.5. Collagen Photocrosslinking

For crosslinking, collagen sheets loaded with 0.01% (w/v) RB in PBS were exposed for 10 min (from one side) to green light (λ = 565 nm) using a mounted LED (M565L3, Thorlabs, Bergkirchen, Germany). The samples were placed 2 cm from the light source so that an irradiance of 45.8 mW/cm^2^ was achieved. The irradiance was measured with a photo power sensor connected to a power and energy meter console (S120VC, PM100D, Thorlabs, Bergkirchen, Germany). In order to prepare collagen laminates, two collagen sheets were first loaded with PBS. Then, 20 µL of 0.1% (w/v) RB in PBS was pipetted onto one collagen sheet and spread on the surface. After placing the second collagen sheet on top of the first, the stack was exposed to green light for 10 min as described for single sheets.

### 4.6. Transmitted Light Microscopy and Scanning Electron Microscopy

Collagen sheets were examined via TLM using polarized light and off-axis illumination (LeicaDM4 M, Leica Microsystems GmbH, Wetzlar, Germany) and SEM (EVO^®^ MA 15, Carl Zeiss AG, Oberkochen, Germany). Samples for light microscopy were conditioned for 24 h in PBS prior to examination. For SEM analysis, dry collagen samples were attached on adhesive carbon tape and covered with a 2 nm gold layer by vapor deposition.

### 4.7. Analysis of Collagen Thickness

Thickness was measured at RT and 37 °C for unmodified and modified collagen sheets, as well as for collagen laminates. All samples were first conditioned for 24 h in PBS and then analyzed via height gauge (DIGIMAR CX1-DX1, Mahr, Göttingen, Germany). For this purpose, the samples were subjected to a force of 1 N ± 0.2 N exerted on a circular area with a diameter of 1 cm. The probing speed and debouncing time were set to 3 mm/s and 3 s, respectively.

### 4.8. Cell Culture

Primary osteoblasts and muscle cells from different donors were used to test the biocompatibility of the sheets. The use of residual materials was approved by the ethics committee of the Landesärztekammer Rheinland-Pfalz (State Authorization Association for Medical Issues) in agreement with the university clinic. Human osteoblasts were isolated according to a previously published protocol [64,65]. Briefly, attached fibrous and fat tissue was carefully removed from bone fragments obtained during hip replacements. Fragments were rinsed in PBS (Gibco^®^ Invitrogen™ Life Technologies, Carlsbad, CA, USA) and digested with collagenase type IV (Sigma Aldrich^®^ GmbH, St. Louis, MO, USA) for 45 min at 37 °C. After washing with PBS, three ∼2 mm^3^ bone specimens were placed in 6-well plates (Becton-Dickinson, Heidelberg, Germany) and cultured in DMEM/F12 (Biochrom, Berlin, Germany) supplemented with 10% fetal calf serum (FCS) (PAA Lab, Pasching, Austria), 100 U/mL penicillin and 100 μg/mL streptomycin sulphate. Cells were incubated in humidified atmosphere (5% CO_2_, 37 °C), and the medium was replaced twice per week. Muscle cells were isolated from lumbar tissue according to a previously published protocol [66]. For this purpose, the perimysium, cell debris and unspecific tissue were removed, and the muscle samples were hackled into 1 mm^2^ fragments. The tissue was washed with PBS and incubated in collagenase (type 2, 470 U/mL, Worthington Biochemical Corporation, Lakewood, CO, USA) in DMEM/F-12 for 1 h at 37 °C in a water bath. After centrifugation (7 min, 1600 rpm), the supernatant was discarded, and the sample was incubated with trypsin/EDTA (0.25%/ 0.02%, 20 min; Biochrom GmbH, Berlin, Germany). Medium was added to stop trypsinization, and samples were filtered through a cell filter (70 µm). After centrifugation (1400 rpm, 5 min) the pellet was resuspended in media, and the cell suspension was cultured for 2 h in the incubator. Subsequently, medium-containing muscle cells were transferred into another cell culture flask coated with collagen (collagen type 1 (CORNING^®^, Discovery Labware, Amsterdam, The Netherlands)/PBS (1:100 (v/v)).

### 4.9. AlamarBlue^®^ Assay

The cell compatibility of the sheets was tested with the alamarBlue^®^ assay. For this purpose, round sheets were equilibrated for 2 h in PBS, sterilized for 45 min under UV light and placed in a 48-well plate for drying overnight. A total of 2500 cells/well were seeded onto the sheets, and the alamarBlue^®^ assay was performed on days 1, 7 and 10. To this end, cells were incubated for 4 h at 37 °C with 320 µL of a 10% alamarBlue^®^ solution. Subsequently, 3 × 100 µL of the supernatant was transferred into a 96-well plate, and the absorbance (560/600 nm) was measured. As controls, cells were seeded onto untreated 48-well plates. To exclude an interaction of alamarBlue^®^ and RB, a second internal control was used for which the results of the sheets without cells were subtracted from the data obtained with the cell-loaded sheets.

### 4.10. Release of Vancomycin

Collagen sheets were loaded with 1 mg of vancomycin in PBS containing 0.01% (w/v) RB and modified by RGX. Unmodified collagen sheets loaded with 1 mg of vancomycin in PBS were used as control. To investigate the release of vancomycin, collagen sheets were used directly after treatment loading and incubated in a 24-well plate in 1 mL of PBS at either RT or 37 °C. Samples were taken after 30 min, 1 h, 2 h, 4 h, 8 h and 24 h by agitating and withdrawing the entire supernatant and adding 1 mL of fresh PBS to the collagen sample. The samples were analyzed by reversed-phase HPLC on a Shimadzu LC20-AD system, SIL-20AC autosampler, SPD-M20A photodiode array detector and a C18 250 × 4.6 mm Synergi™ 4 µm Fusion-RP 80 Å column (Phenomenex, Aschaffenburg, Germany). Then, 100 µL of the supernatant was injected onto the column by autosampler. After washing for 20 min with eluent A (95% water, 5% acetonitrile, 0.1% TFA), the concentration of eluent B (5% water, 95% acetonitrile, 0.1% TFA) was steadily increased from 0% to 90% over 50 min at a flow rate of 0.5 mL/min. Absorption was monitored at 280 nm for vancomycin and at 220 nm for collagen. Vancomycin eluted after a retention time of 41 min. The amount of released vancomycin was calculated from a calibration curve of vancomycin from 0 to 1 mg/mL.

## 5. Conclusions

Commercially available collagen sheets can be crosslinked by rose bengal and green light crosslinking (RGX) to tailor their properties. While the effect of RGX on dense collagen materials such as those of Collagen Solutions and Viscofan is small to not measurable, the non-crosslinked fragile Atelocollagen sponge was stabilized by RGX, resulting in a 20% reduced swelling degree and 7.4 to 32.7 times increased resistance towards mechanical pressure. Primary osteoblasts and muscle cells did not proliferate on Viscofan collagen samples independent of treatment. On Collagen Solutions and Atelocollagen samples, proliferation of these cells was improved after RGX due to the increase in collagen rigidity. At the same time, RGX had no impact on the release of vancomycin. Combination of commercially available materials and modification by RGX is a means to tailor mechanical stability and cell compatibility. For this purpose, collagen sheets can be crosslinked by RGX.

## Supplementary Materials

Supplementary materials can be found at https://www.mdpi.com/1422-0067/21/19/7408/s1. Figure S1. Swelling degree of unmodified collagen sheets at 37 °C and RT after 2 h, 4 h and 24 h in relation to their dry weight (100%). Figure S2. Swelling degree of unmodified collagen sheets, collagen sheets loaded with 0.01% RB exposed to standard laboratory illumination (0 min, 0.01% RB) and collagen sheets loaded with PBS exposed to green light (10 min, 0% RB) after 2 h at 37 °C. Figure S3. Thickness (measured by height gauge under force application) of unmodified collagen sheets and “as received” at (A) 37 °C and (B) RT as measured by height gauge under force application. Figure S4. Thickness (measured by height gauge under force application) of unmodified collagen sheets, collagen sheets loaded with 0.01% RB exposed to standard laboratory illumination (0 min, 0.01% RB) or protected from stray light (0 min, 0.01% RB, dark), and collagen sheets loaded with PBS exposed to green light (10 min, 0% RB) at 37 °C and RT. Samples were conditioned in PBS for 24 h before measurement.

## Abbreviations

DSC	Differential scanning calorimetry
PBS	Phosphate-buffered saline
RB	Rose Bengal
RGX	Rose bengal and green light collagen crosslinking
SEM	Scanning electron microscopy
TLM	Transmitted light microscopy
