# Supplementary material for: Rose Bengal Crosslinking to Stabilize Collagen Sheets and Generate Modulated Collagen Laminates
Source: Int J Mol Sci. 2020 Oct 8;21(19):7408. doi: 10.3390/ijms21197408 (PMC7582313; doi:10.3390/ijms21197408)
Supplement: Supplementary file 1 [file ijms-21-07408-s001.pdf]

Article

Rose Bengal Crosslinking to Stabilize Collagen Sheets and Generate Modulated Collagen Laminates

Stefanie Eckes [1], Joy Braun [2] Julia S. Wack [1], Ulrike Ritz [2], Daniela Nickel [3,*] and Katja Schmitz [1,*]

[1] Clemens-Schöpf-Institute of Organic Chemistry and Biochemistry, Technical University of Darmstadt, Darmstadt, Germany; stefanie.eckes@tu-darmstadt.de (S.E.); wack.juliasusanne@googlemail.com (J.S.W.)
[2] Department of Orthopaedics and Traumatology, BiomaTiCS, University Medical Center, Johannes Gutenberg University, Langenbeckstraße 1, 55131 Mainz, Germany; joybraun@uni-mainz.de (J.B); ritz@uni-mainz.de (U.R.)
[3] Berufsakademie Sachsen - Staatliche Studienakademie Glauchau, University of Cooperative Education, Kopernikusstraße 51, 08371 Glauchau, Germany
* Correspondence: katja.schmitz@tu-darmstadt.de; Tel.: +49-(0)6151 16-21015 (K.S.) and daniela.nickel@ba-sachsen.de; Tel: + 49-(0)3763 173-131 (D.N)

Supporting Information

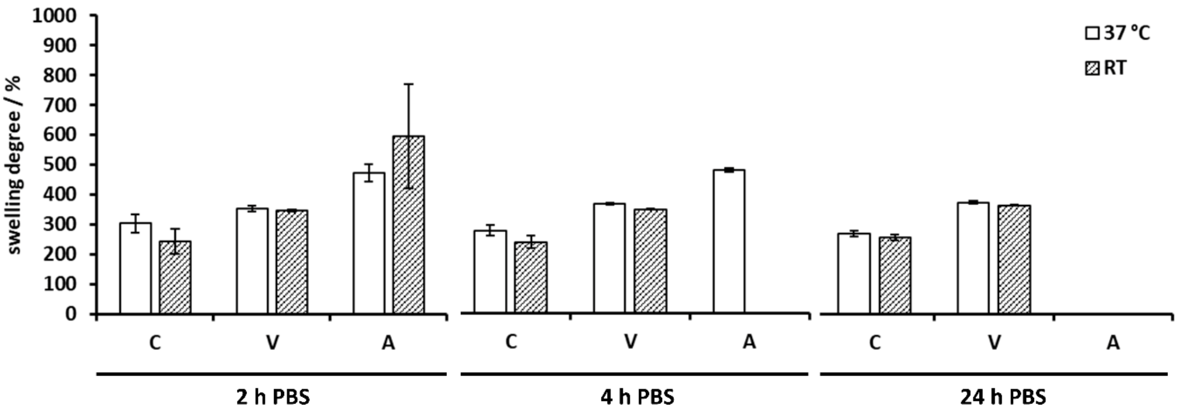

Figure S1. Swelling degree of unmodified collagen sheets at 37 °C and RT after 2 h, 4 h and 24 h in relation to their dry weight (100 %). C: Collagen Solutions, V: Viscofan and A: Atelocollagen. Unmodified Atelocollagen samples were destroyed during 2 h and 4 h measurements so that no results were obtained for 4 h at RT and 24 h.

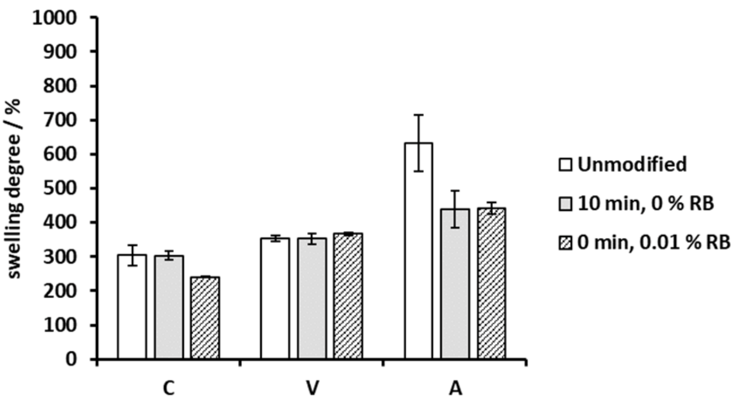

Figure S2. Swelling degree of unmodified collagen sheets, collagen sheets loaded with 0.01 % RB exposed to standard laboratory illumination (0 min, 0.01 % RB) and collagen sheets loaded with PBS exposed to green light (10 min, 0 % RB) after 2 h at 37 °C. C: Collagen Solutions, V: Viscofan and A: Atelocollagen.

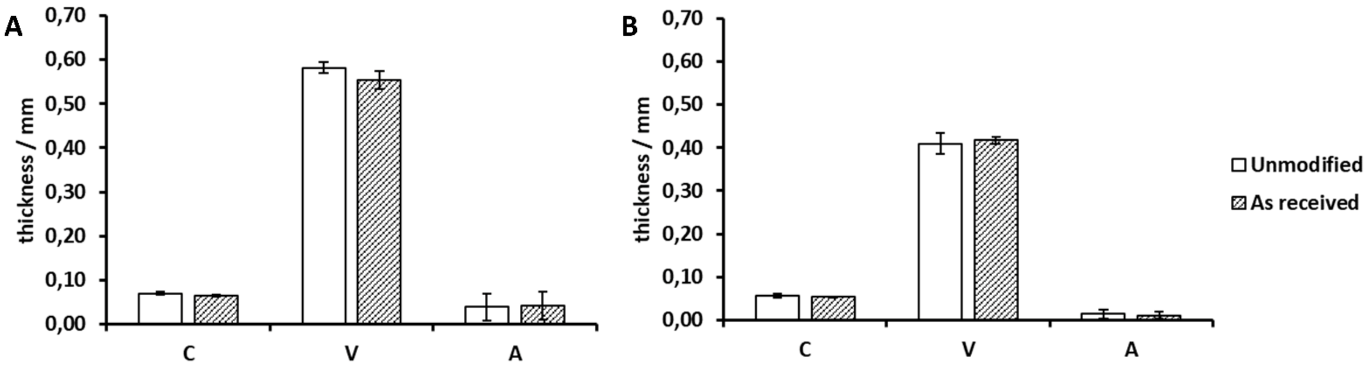

Figure S3. Thickness (measured by height gauge under force application) of unmodified collagen sheets and “as received” at (A) 37 °C and (B) RT as measured by height gauge under force application. Samples were conditioned in PBS for 24 h before measurement. C: Collagen Solutions, V: Viscofan and A: Atelocollagen.

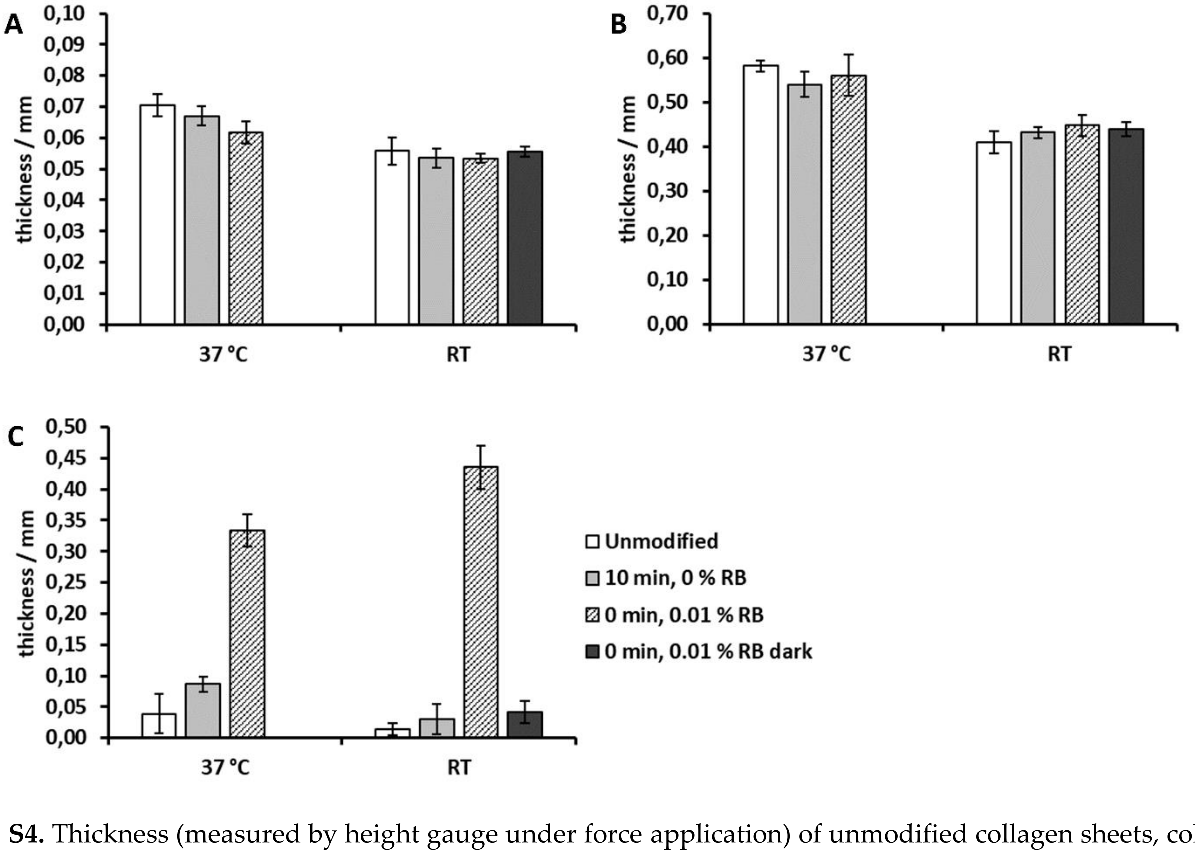

Figure S4. Thickness (measured by height gauge under force application) of unmodified collagen sheets, collagen sheets loaded with 0.01 % RB exposed to standard laboratory illumination (0 min, 0.01 % RB) or protected from stray light (0 min, 0.01 % RB, dark), and collagen sheets loaded with PBS exposed to green light (10 min, 0 % RB) at 37 °C and RT. Samples were conditioned in PBS for 24 h before measurement. (A): Collagen Solutions, (B): Viscofan, (C): Atelocollagen.
